# Prognostic Difficulties in Atypical Pneumonia

**Published:** 1910-04-09

**Authors:** John Broadbent

**Affiliations:** Physician to Out-Patients, St. Mary's Hospital.


### Hospital Clinics.

### PROGNOSTIC DIFFICULTIES IN ATYPICAL PNEUMONIA.

By Sir JOHN BROADBENT, Bart., M.D., F.R.C.P.; Physician to Out-Patients, St. Mary's Hospital.

(Read before the Medical Society of London, February 28, 1910.)

In a paper read before this Society in 1908 on "The Pneumococcus Infections," Professor Osler drew attention to "the enormous increase in the death-rate from that most important of pneumococcus infections, pneumonia," and emphasised the necessity for further study of this disease, more especially in relation to preventive measures. Amongst other points he raised as worthy of consideration was the question as to whether a septicæmia precedes the development of a local lesion, and that of the frequency and variety of clinically atypical forms. I have thought, therefore, that it would be of interest to bring before the Society some cases bearing on these two important questions.

### Atypical Cases.

The atypical cases are in adults, I think, met with chiefly in association with influenza. The insidious onset, the delay in the appearance of physical signs, the intermittent pyrexia, may at the outset render the diagnosis obscure, while the absence of a definite crisis, the continued pyrexia lasting, it may be, some weeks, with progressive loss of flesh, will render the prognosis a matter of considerable difficulty; for in some cases empyema, or malignant endocarditis, may develop, whilst in others, after a prolonged period of septicæmia, recovery takes place without any further complication.

In children the tendency in apical pneumonia to the supervention of symptoms suggestive of cerebrospinal meningitis may cause undue alarm, or the vagaries of a prolonged attack of lobular pneumonia may lead to a suspicion of pulmonary tuberculosis. Broncho-pneumonia complicating enteric fever in children may also be misleading, and mask the symptoms of the latter affection, which in children often runs an atypical course. For instance, I recollect one case in a child admitted to St. Mary's Hospital in which the irregularity of temperature from the presence of broncho-pneumonia and the absence of spots or any classical signs of enteric led to an error in diagnosis, and it was not till a typical relapse, accompanied by the appearance of typhoid spots ten days after the temperature had fallen to normal, that it was recognised that the initial illness had been enteric complicated by broncho-pneumonia, and not the latter affection alone. I do not, however, propose to discuss further the difficulties in prognosis in atypical pneumonia in children.

### Some Illustrative Cases.

I will now proceed to lay before you brief notes of three atypical cases of pneumonia which occurred during a severe epidemic of influenza, in which the temperature was extremely irregular throughout. No crisis occurred, and the pyrexia was prolonged, lasting in one case as long as five weeks before recovery took place.

The first case was that of a man aged 42, admitted to St. Mary's Hospital on April 24, 1900, under Dr. Lees, who has kindly given me permission to make use of this and the following two cases. The patient was at work till April 21, and felt quite well on going to bed on the 20th. He woke up on the morning of the 21st with bad headache, and was perspiring freely. In the afternoon he had severe pain in the left side of the chest, worse on taking a deep breath. He became very short of breath in the evening and could not sleep. He stayed at home on the 22nd and 23rd, and was admitted on the 24th to St. Mary's Hospital.

He was a well-nourished, strong, well-developed man, with cyanosed lips, and pulse 126, resp. 42, temp. 104° F. There was dulness over the left base behind up to the angle of the scapula, with absence of vocal resonance and fremitus, and very poor air entry. A few pleuritic crackles were audible over the back. There was dulness in the left axilla, with bronchial breathing in the upper part and bronchophony. The tongue was coated with a thick white fur, and he was very restless and delirious.

On the 26th there was dulness over the whole of the left base in front and behind, with bronchial breathing over the back as well as in the axilla. On April 27 there was dulness also over the lower lobe of the right lung with deficient air entry, and bronchial breathing was audible over a small area in the axilla. The temperature was extremely irregular, the pulse rapid and feeble, and the loss of strength very pronounced. He was very delirious throughout. The physical signs remained much the same to the end, and he died on May 4, on the twelfth day after admission to the hospital.

At the autopsy the lower lobe of the left lung was slightly adherent to the chest wall by a thick layer of sticky recent lymph. It was intensely congested and œdematous, dripping with blood, soft to the touch, and plum-coloured on section. Its condition did not in the least resemble that of the red or grey hepatisation usually met with in fatal pneumonia. Portions cut out just sank in water. The right lung was also intensely congested, and the lower lobe presented the same characteristics as that of the left lung. Sections showed extreme congestion, numerous hæmorrhages, and partial collapse, with very little inflammatory reaction.

### The Pathological Findings.

The pathological findings in this case are interesting as throwing some light on the probable condition of the lungs in the two following cases in which the pneumococcal infection took the form of a profound and prolonged septicæmia from the outset. It would appear that in this group of cases, though the lung was primarily attacked, the absence of the usual inflammatory reaction which occurs in typical pneumonia, allowed of the pneumococci to flourish in a suitable nidus of congested tissue for an indefinite period, so that instead of the affection terminating by crisis, a prolonged septicæmia resulted.

### Another Atypical Case.

The second case was that of a boy aged 14, admitted to St. Mary's Hospital under Dr. Lees, on March 16, 1900, for pain in right side of chest. He said he was well till the 13th, three days before admission, when he felt ill and shivery and had a troublesome cough. On the 14th he had a rigor and severe pain in the head, and felt so ill that he went home and then stayed in bed till the 16th, when he was admitted to St. Mary's. On admission he had temp. 100° F., pulse 132, resp. 48; there was dulness in the left axilla, with pleuritic crackles, and dulness behind with deficient air entry and friction sounds over the base. On the right side there was a patch of dulness just below the angle of the scapula, with tubular breathing, bronchophony, impaired resonance, and deficient air entry behind. The tongue was coated with a thick white fur.

Both bases became completely dull behind, and tubular breathing developed over the dull area. The dulness and tubular breathing over both bases, and the pyrexia persisted for four weeks, and the boy lost flesh rapidly. An exploring needle inserted on three or four occasions failed to discover any pus. Tuberculosis was then suspected, but no tubercle bacilli were found in the sputum. At length, on April 27, after six weeks, the temperature fell to normal and remained normal thereafter. He then put on flesh rapidly and gained strength, and was discharged cured on May 7, ten days later.

### A Third Case.

The third case was that of a man aged 24, admitted to St. Mary's Hospital on May 4, 1900, under Dr. Lees. A fortnight before he felt ill, but kept on with his work till May 2, when he felt so ill he could not get out of bed. No rigors or pain in the side had been experienced. On admission he had temp. 101° F., pulse 96, resp. 28. He was a well-nourished man, slightly cyanosed. There was dulness in the right axilla and over the right lower lobe behind, with deficient air entry and pleuritic crackles. Tubular breathing was heard on auscultation over the dull area in right lower behind, but not in the axilla. There was also a small area of dulness at the base of the left lower lobe with pleuritic crackles and bronchial breathing. This very soon cleared up.

The dulness over the right lower lobe with the tubular breathing and the pyrexia persisted for nearly five weeks, when the temperature fell to normal and he rapidly regained flesh and strength. On his discharge on June 23 there was still dulness at the right base, with poor air entry and deficient vocal resonance. There was also a small area over which bronchial breathing was audible, but the patient seemed quite well and strong, and the temperature had been normal for a fortnight before he was discharged.

These three cases illustrate the difficulties that may be met with in prognosis in atypical cases of pneumonia. The temperature throughout was of an irregular type, and suggested the possibility of empyema, but the repeated exploratory punctures in the cases that recovered, and the post-mortem examination in the fatal case negatived this. As already stated, from the pathological evidence in the fatal case it would seem that in all probability there was little reaction on the part of the tissues and the collapsed and congested lung formed a suitable nidus for the growth of the pneumococcus which thus gave rise to a general septicæmia of prolonged duration.

### An Irregular Case.

In the following case, on the contrary, with a similar irregular temperature chart an empyema rapidly developed. Edward J., 17, a porter, was admitted to the Hampstead Hospital on August 13, 1909. The patient stated that he was well till the morning of August 13, and was filling a coal bucket at a flat when he fell ill and shivering, had a bad headache, and vomited. He went home and felt very drowsy, was sick again in the evening, and became feverish and thirsty and complained of pain in the right side. There was no cough. He was seen by a doctor, who sent him into the hospital as possibly a case of sunstroke.

He was a well-nourished youth, face pale with cheeks flushed. Temp. 104°, pulse 136, resp. 32. He was drowsy and somewhat delirious at night. The chest was resonant all over on both sides. There were no adventitious sounds. The tongue was thickly coated with white fur; abdomen normal.

Next day temp. was 101° in the morning, and patient seemed better; pulse 104, resp. 48. Resonance was normal on both sides, and there were no adventitious sounds. On August 15 temperature was normal in the morning, but the pulse rate was still 100 and resp. 36. Herpes was present on the lips and at the angle of mouth. There was slightly diminished movement of right side of chest and diminished air entry. Resonance slightly impaired over right base. No adventitious sounds. Temperature rose to 101 in the afternoon, and the patient became very restless and delirious.

August 16.—Temp. 98.2 in morning, pulse 80, resp. 36.

Dulness over the right base behind below the angle of scapula extending forwards only as far as the posterior axillary line. Impaired vocal resonance and fremitus over the area with marked diminution of air entry. Just below and slightly external to the angle of the scapula is a small patch about 2 in. by 2 in. over which definite tubular breathing is heard with bronchophony. No crepitations audible, over front of right lung is high-pitched Skodaïc resonance.

August 17.—Temp. 99 in morning, pulse 84, resp. 32. There is a cough with expectoration of some blood-stained mucus. Physical signs in the lungs not appreciably altered. On August 19 there was dulness and tubular breathing over the upper part of the right lung behind, with increased vocal resonance and fremitus. Dulness and poor entry, with absence of V.F. and V.R. below the angle of the scapula as far as the mid-axillary line, where crepitations are audible. Temp. 100.2° in morning, 101.4° at night.

August 23.—The patient seems better. The tubular breathing over the back of the upper half of the right lung has gone and is replaced by moist sounds. Resonance over this area improved, no actual dulness, but complete dulness below the angle of the scapula behind with absence of V.F. and V.R.

August 27.—Right lung clearing up except at the base, where dulness and signs of friction persist. Moist sounds are audible over the upper half and in the axilla with good air entry.

On September 2, as the pyrexia persisted and the patient was perspiring at night, it was thought probable that there was an empyema. An exploring needle was inserted by the house physician in the right base, but he failed to obtain pus.

On September 3 an exploring needle was again inserted on the same side two inches below angle of scapula and two inches external, and drew off pus.

The empyema was evacuated, and the man was put back to bed, and seemed to be all right; but ten minutes later he had a sudden syncopal attack, which proved fatal in spite of all efforts to restore him.

### Findings at the Autopsy.

At the autopsy the heart and pericardium, brain and meninges were normal. The empyema had been thoroughly evacuated. The left lung was intensely congested, hæmorrhagic, and partially collapsed, but there was no pneumonic consolidation, and its condition very much resembled that of the lung in the fatal case recorded above. The only explanation for his sudden and unexpected death would seem to be that the shock of the operation on top of the profound and prolonged septicæmia had been too severe for him to withstand in his enfeebled condition. In this case it was three days from the onset of the symptoms and pyrexia before any definite physical signs in the chest supervened, and it would appear to prove, as suggested by Professor Osler, that pneumococcal septicæmia may precede the development of local inflammatory reaction in the lungs or pleura. The toxæmia in this case, as in the three preceding ones, was a predominant feature throughout. From the character of the pyrexia at the outset and the similarity of the physical signs when they developed I thought that this case was analogous to the three cases already described, but, contrary to expectation, an empyema developed.

In contrast to these cases, and illustrating the difficulties in diagnosis as well as prognosis is a case of protracted pyrexia which simulated in all respects an attack of influenzal pneumonia at the outset, but which eventually proved to be pulmonary tuberculosis. The following are brief notes of the case:

James R., aged 32, was admitted to the Hampstead General Hospital July 13, 1909. He was well and at work till a month before admission, when he lost his appetite, and had shivering attacks and some pyrexia, thought to be due to an attack of influenza. On July 8 he had some pain in the right side of the chest, worse on taking deep breath or coughing. He then developed a troublesome cough, and brought up some rusty sputum, and at night his temperature rose to 105°.

On admission to the hospital on July 10 he was pale and very short of breath. Temperature 101.2°, pulse 72, resp. 28. There was dulness and tubular breathing over the right base behind from line one inch above angle of scapula, the base extending forward to the mid-axillary line. Over the dull area there was increased vocal resonance fremitus. On July 17 a friction rub developed at the left base, the condition of the right lung remaining much the same.

The irregular pyrexia persisted, and the patient sweated profusely at night. A needle was inserted into the right base on three occasions with negative result. The physical signs in the lungs remained much the same, and though a blood culture taken on August 28 proved sterile, it was thought it might be a case of delayed resolution with pneumococcal septicæmia, as no tubercle bacilli were found in the sputum.

As he continued to lose flesh and the night sweats and pyrexia persisted, it was thought it might be a case of acute pneumonic phthisis, especially as some crepitation became audible over the apex of the right lower lobe. Both Von Pirquet's and Calmette's reaction for tuberculosis were positive, which seemed to confirm the diagnosis.

He was then put out on the balcony all day in the open air, as far as weather permitted, and improved very markedly, the temperature falling practically to normal during the first three weeks of October, while he gained steadily in weight. The weather conditions and hospital arrangements did not allow of efficient open-air treatment, and towards the end of October the temperature again became irregular. On November 9 he was discharged, after 17 weeks in hospital. On admission his weight was only 7 st. 6 lb., but on discharge it was 8 st. 9 lb., and his general condition was much improved. There was still dulness and tubular breathing over the base of the right lung as far as the mid-axillary line, with a patch of dulness in the left axilla and a few crepitations at the apex of the lower lobe.

He went down into the country to Devonshire, and at the end of December reported that he was gradually getting worse. I have had no further news of him since.

### A Case of Pneumococcal Septicæmia.

I have yet one more case of pneumococcal septicæmia to bring before your notice in which empyema, malignant endocarditis, and suppurative meningitis developed.

The patient was a man aged 48, who was admitted to St. Mary's Hospital, August 26, 1909, under my care while in charge of the wards for Dr. Phillips, with the history that three weeks ago, on August 5, he was taken ill with shivering, fever, and pain in the chest. He saw a doctor, who told him he had pneumonia and must stay in bed, where he remained till date of admission to the hospital.

He was a fairly well-nourished man, looked very ill, lips and cheeks cyanosed. Temp. 99.2°, pulse 96, resp. 26. On the right side the murmurs are better than on the left. Left front high-pitched Skodaïc resonance. Dulness over left back below the angle of scapula extending forwards to posterior axillary line. Over this area absence of vocal resonance and fremitus and poor air entry. On the right side resonance was normal: rhonchi and sibili were audible over front and back. The pulse was regular in force and frequency, collapsing in type, suggestive of aortic regurgitation. Marked throbbing of carotids. Apex beat not visible nor palpable. Cardiac dulness extended one inch to right of sternum and one inch to left of vertical nipple line. Soft systolic and diastolic murmur audible in the fifth space in the vertical nipple line. Apex beat not palpable, first sound very poor. With difficulty, owing to bronchitic sounds, soft diastolic murmur was heard just to the left of the sternum in the third and fourth spaces. No murmur audible at the aortic cartilage in the second right intercostal space. The temperature in the evening of the 26th rose to 103.4. On August 27, as the physical signs were suggestive of empyema, an exploring needle was inserted over the base of the right lung in three different situations, but no pus was withdrawn. The temperature in the morning was 99.2, but rose in the evening to 103.8. Urine: s.g. 1022. Small amount of albumen. Leucocyte count 25,000 leucocytes to cub. mm. The intermittent pyrexia persisted, and he got steadily worse, and malignant endocarditis was diagnosed. Cultures taken from blood drawn from the median basitic vein, however, proved sterile. On September 1 he developed right hemiplegia with aphasia, which pointed to embolism, and he died the same night.

### Findings at the Autopsy.

At the post-mortem examination there was an empyema at the left base, with thick viscid pus. The left lung was congested, collapsed, and hæmorrhagic. There was a large brittle fungating vegetation on the aortic valve, due to malignant endocarditis of recent origin. There was suppurative meningitis, with lymph over the vertex and the left side of the brain, chiefly over the Rolandic area. No embolism was found after careful search in the middle cerebral and its branches.

This was an interesting case, and illustrates three of the possible sequels of pneumonia. On admission, from the physical signs in the left lung and pyrexia, empyema was diagnosed; but as a needle failed to withdraw pus after three explorations, presumably owing to the viscid nature of the pus, it was thought that the lung condition might be similar to that in the first three cases described, in which there was presumably a layer of lymph or thickened pleura with collapsed lung beneath. The presence of a diastolic murmur, with absence of any hypertrophy of the left ventricle, together with marked cardiac dilatation and septicæmia, led to the diagnosis of malignant endocarditis of recent origin, which the autopsy confirmed. The onset of aphasia, with paralysis of the right arm, seemed to point to embolism of the left middle cerebral artery or one of its branches; but careful search failed to reveal any embolus, so that the hemiplegia was presumably due to the suppurative meningitis, the exudation being most abundant over the Rolandic area.

### Some Conclusions.

I think the above cases are not without interest as instances of "clinically atypical pneumonia," and as illustrating the septicæmic character that pneumococcal infections may assume, even when the lung is primarily attacked. The three cases first described, and others of the same type, of which I have notes, occurred during a serious influenza epidemic, and it is possible that influenza may be in many instances responsible for the atypical septicæmia type of pneumonia. The most important ætiological factor, in the light of the pathological evidence referred to above, would seem to be the absence of the usual inflammatory reaction on the part of the lung when attacked by the pneumococcus. This lowered resistance might well be due, in many instances, to an attack of influenza immediately preceding. The collapsed and congested condition of the lung with the thick layer of overlying lymph found post-mortem in the fatal case, tends, I think, to throw some light on the cause of the delayed resolution such as occurred in cases two and three, and is met with in analogous cases of influenzal pneumonia.

